# Mapping the Lymphatic Drainage Pattern of Esophageal Cancer with Near-Infrared Fluorescent Imaging during Robotic Assisted Minimally Invasive Ivor Lewis Esophagectomy (RAMIE)—First Results of the Prospective ESOMAP Feasibility Trial

**DOI:** 10.3390/cancers15082247

**Published:** 2023-04-12

**Authors:** Dolores T. Müller, Lars M. Schiffmann, Alissa Reisewitz, Seung-Hun Chon, Jennifer A. Eckhoff, Benjamin Babic, Thomas Schmidt, Wolfgang Schröder, Christiane J. Bruns, Hans F. Fuchs

**Affiliations:** 1 Department of General, Visceral, Cancer and Transplant Surgery, University of Cologne, Kerpener Str. 62, D-50937 Cologne, Germany; 2 Center for Esophagogastric Cancer Surgery Frankfurt, St. Elisabethen Hospital Frankfurt, D-60487 Frankfurt am Main, Germany

**Keywords:** esophageal cancer, robotic-assisted minimally invasive esophagectomy (RAMIE), near-infrared fluorescence imaging, indocyanine green (ICG), lymph node mapping

## Abstract

**Simple Summary:**

While the sentinel lymph node concept is routinely applied in other surgical fields, no established and valid modality for lymph node mapping for esophageal cancer surgery currently exists. Near-infrared light fluorescence (NIR) using indocyanine green (ICG) has been recently proven to be a safe technology for peritumoral injection and consecutive lymph node mapping in small cohorts. The aim of this study was to identify the lymphatic drainage pattern of esophageal cancer during robotic-assisted minimally invasive esophagectomy (RAMIE) and to correlate the intraoperative images with the histopathological dissemination of lymphatic metastases. *n* = 20 patients were included in the study, and feasibility and safety for the application of NIR using ICG during robotic-assisted minimally invasive RAMIE were shown. We conclude that RAMIE can be safely combined with NIR to detect lymph node metastases. Further analyses in our center will focus on the ICG-positive tissue as well as quantification and long-term follow-up data.

**Abstract:**

While the sentinel lymph node concept is routinely applied in other surgical fields, no established and valid modality for lymph node mapping for esophageal cancer surgery currently exists. Near-infrared light fluorescence (NIR) using indocyanine green (ICG) has been recently proven to be a safe technology for peritumoral injection and consecutive lymph node mapping in small surgical cohorts, mostly without the usage of robotic technology. The aim of this study was to identify the lymphatic drainage pattern of esophageal cancer during highly standardized RAMIE and to correlate the intraoperative images with the histopathological dissemination of lymphatic metastases. Patients with clinically advanced stage squamous cell carcinoma or adenocarcinoma of the esophagus undergoing a RAMIE at our Center of Excellence for Surgery of the Upper Gastrointestinal Tract were prospectively included in this study. Patients were admitted on the day prior to surgery, and an additional EGD with endoscopic injection of the ICG solution around the tumor was performed. Intraoperative imaging procedures were performed using the Stryker 1688 or the FIREFLY fluorescence imaging system, and resected lymph nodes were sent to pathology. A total of 20 patients were included in the study, and feasibility and safety for the application of NIR using ICG during RAMIE were shown. NIR imaging to detect lymph node metastases can be safely performed during RAMIE. Further analyses in our center will focus on pathological analyses of ICG-positive tissue and quantification using artificial intelligence tools with a correlation of long-term follow-up data.

## 1. Introduction

According to national and international guidelines, two-field lymphadenectomy depicts the current standard during transthoracic esophagectomy for esophageal cancer. A highly individualized and more targeted lymphadenectomy, however, could improve a patient’s oncological outcome, as a more extensive lymphadenectomy than the regular D2 or 2-field approach could be performed if needed [1,2]. The sentinel lymph node concept is routinely applied in other surgical fields. However, no established and valid modality for lymph node mapping for esophageal cancer surgery exists. Near-infrared light fluorescence (NIR) using indocyanine green (ICG) has been recently proven to be a safe technology for the visualization of vascular or biliary structures in gastrointestinal surgery, but also for lymphatic mapping, offering improved visibility [3,4]. In addition, Xiong et al. published a meta-analysis of the potential of ICG to identify sentinel lymph nodes in breast, colon, gastric, lung, prostate, cervical, endometrial, esophageal, and oral cancer with a pooled specificity of 100% [5]. Recent studies have shown its various applications in esophageal surgery, ranging from visualization of gastric conduit perfusion to thoracic duct identification [6,7,8]. Furthermore, Schlottmann et al. have proven the feasibility of a peritumoral injection of ICG and consecutive lymph node mapping using NIR in 9 patients undergoing esophagectomy at a single center [9]. An evaluation of a robotic minimally invasive approach (RAMIE) in addition to a systematic correlation with histopathology has yet only been done in small case series [10].

The aim of this study was to identify the lymphatic drainage pattern of esophageal cancer during RAMIE and to correlate the intraoperative images with the histopathological dissemination of lymphatic metastases.

## 2. Materials and Methods

### 2.1. Patients

Patients with clinically advanced stage squamous cell carcinoma or adenocarcinoma of the esophagus and gastroesophageal junction Siewert Type I or II (T1b–T4, N0–N3) using the International Union Against Cancer [UICC] TNM Classification 7th edition undergoing a robotic-assisted minimally invasive Ivor Lewis esophagectomy at our Center of Excellence for Surgery of the Upper Gastrointestinal Tract were prospectively included in this study. All patients provided written informed consent. Prospective data collection was conducted with approval from the institutional review board at the University of Cologne, Germany (IRB reference 19-1463).

### 2.2. Preoperative Oncological Staging

Patients received, as a preoperative oncological staging, an esophagogastroduodenoscopy (EGD) by an experienced gastroenterologist or endoscopist and biopsy for histopathological analysis, endoluminal ultrasound (EUS), computed tomography (CT) of the abdomen and thorax, and FDG-PET/CT (optional). The complete preoperative workup further included a physical examination, medical history, demography, vital signs, body weight, electrocardiogram, and standard laboratory tests. In addition, cardiology review/echocardiography (ejection fraction > 50%) and/or pulmonary function tests (FEV1 > 1.5 L) were mandatory. For patients with locally advanced adenocarcinomas/squamous cell carcinomas, multimodal treatment is now standard care. The post-neoadjuvant diagnostics included a CT scan and esophagogastroduodenoscopy (EGD). We have published the standardized treatment pathway for patients with resectable esophageal cancer applied at our center before [11]. After completion of baseline assessments, either after restaging or if a primarily surgical approach was chosen after staging, a check and validation of inclusion and exclusion criteria for the study were performed.

### 2.3. Surgical Approach

Following restaging, typically 4–6 weeks after neoadjuvant therapy, patients received a robotic-assisted transthoracic esophagectomy with a high intrathoracic anastomosis and reconstruction using a gastric conduit (RAMIE procedure). Risk assessment was performed preoperatively using a standardized and validated risk scoring system [12]. A minimally invasive approach was applied in all patients, with the abdominal segment performed either laparoscopically or robotically. The thoracic part was performed robotically in all patients. We have previously published a detailed report on our operative technique [13]. If suitable, patients with an adenocarcinoma of the gastroesophageal junction Siewert Type II were enrolled in the European multicenter CARDIA trial (KS2017-209), which aims to compare the oncological outcome and postoperative quality of life of transthoracic esophagectomy versus transhiatal gastrectomy [14]. Only patients who were randomized for a transthoracic esophagectomy and underwent a RAMIE procedure could be included in this present study.

### 2.4. Lymphadenectomy and Operative Technique RAMIE

The extent of the lymph node dissection comprises the D2 compartment, including, aside from the perigastric lymph nodes, all lymph nodes around the celiac axis, common hepatic artery, left gastric artery, and splenic artery. In line with the German S3 guidelines for gastric cancer, the D2 lymph node dissection should allow an examination of at least 25 lymph nodes [15]. In addition, the major and minor omentum were resected. The esophagectomy was performed utilizing a right transthoracic minimally invasive approach combined with resection of the proximal stomach (GEJ). This always included a 2-field lymphadenectomy of the mediastinal (para-esophageal, subcarinal, para-tracheal, aorto-pulmonary) and abdominal lymph nodes. A gastric conduit was constructed, and continuity was re-established by intrathoracic anastomosis (Ivor-Lewis). Lymph node dissection in the upper mediastinum during transthoracic esophagectomy was performed by the surgeon en-bloc, including paraesophageal lymph nodes, subcarinal lymph nodes, paratracheal lymph nodes, and lymph nodes of the aortopulmonary window. If any lymphatic tissue in the station mentioned before was positive for ICG uptake, this was separately resected and sent to pathology. We aimed to resect at least one ICG-positive lymph node if available. Also, a negative probe of lymphatic tissue was always taken for separate analysis. Through this protocol, the pathologist could individually analyze the different lymph node packages based on ICG uptake status. Each numbered and labeled lymph node was placed in a labeled specimen capsule container and sent to histopathology for Hematoxylin and Eosin staining and further workup. No complete “histopathological map” of every lymph node station was performed in this pilot feasibility trial. It was more the aim of this study to prove technical feasibility.

### 2.5. ESOMAP Protocol

**Figure 1:**
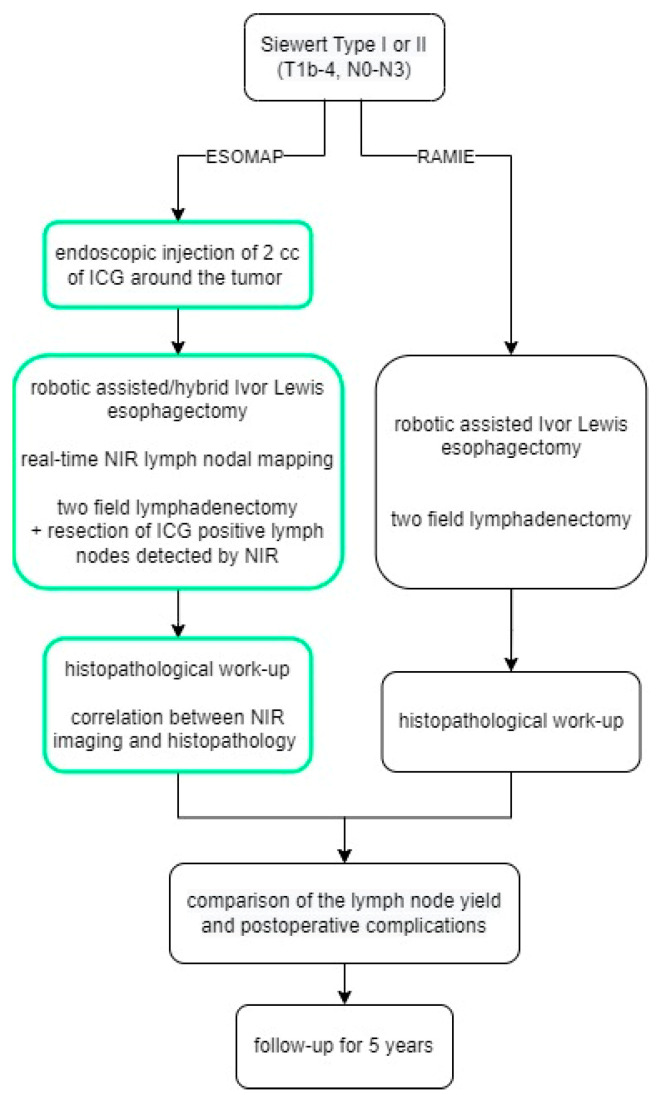
The ESOMAP trial.

Patients were admitted on the day prior to surgery, and an additional EGD with endoscopic injection of the ICG solution (Diagnostic Green LLC, Farmington Hills, MI, USA) around the tumor was performed, as shown by Schlottmann et al. and others before, either on admission day or directly prior to surgery [9,16]. Indocyanine green was prepared immediately before the endoscopic procedure. 25 mg ICG was diluted in 10 mL water to make a 2.5 mg/mL solution, then 0.2 mL of the solution was diluted again in 10 mL water to make a 0.05 mg/mL solution used in the study. Patients then underwent surgery with near-infrared fluorescence lymph nodal mapping the following day. Intraoperative imaging procedures were performed using the Stryker 1688 (Stryker Corporation, Kalamazoo, MI, USA) or the FIREFLY fluorescence (Intuitive Surgical Inc., Sunnyvale, CA, USA) imaging system. NIR was not turned on constantly but rather used on demand to assist with the lymphadenectomy. A final check with NIR turned on was always performed after the completion of D2 lymphadenectomy. Per study protocol, the aim was to resect at least one ICG-positive lymph node per patient. If any ICG-positive tissue was found after completion of the lymphadenectomy, this was resected and sent for a separate analysis. A flow chart summarizing the structure of the study is shown in Figure 1. Surgery was performed using the latest robotic system available (Davinci Xi, Intuitive Surgical, Sunnyvale, CA, USA).

### 2.6. Standardized Follow-Up after RAMIE

All patients undergoing the RAMIE procedure for esophageal cancer are enrolled in a standardized follow-up program at our outpatient clinic. For 5 consecutive years, patients are evaluated using a standardized schedule, including physical examinations, CT scans, EGDs, and tumor markers at specific time points.

### 2.7. Data Analysis and Statistical Evaluation

For the statistical data analysis, patients that underwent the ESOMAP protocol were compared to a standardized cohort of patients that underwent a RAMIE procedure during the same period. Categorical data are presented as numbers and percentages. Continuous data are shown as means and ranges. The Student *t*-test (for continuous variables) and Fisher’s exact test (for nominal or categorical variables) were used for all bivariate analyses. All tests were 2-sided, with statistical significance set to *p* ≤ 0.005.

## 3. Results

A total of 106 patients underwent RAMIE from 2019 to 2021 with a standardized circular stapled anastomosis. Of these, 20 patients received the ESOMAP protocol (ESOMAP group) in addition. Demographic and oncological data of both groups are shown in Table 1. The mean age was 60 years (range 46–72 years) in the ESOMAP group and 63 years (range 47–80 years) in the RAMIE group, *p* = 0.1238. Mean BMI was 25.6 kg/m^2^ (range 18.5–35.4 kg/m^2^) in the ESOMAP group vs. 25.5 kg/m^2^ (range 15.6–35.2 kg/m^2^), *p* = 0.9106.

### 3.1. ESOMAP Protocol/Oncological Outcome

**Figure 2:**
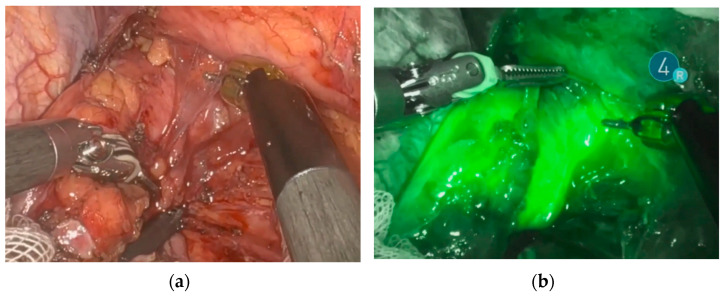
Identification and visualization of the esophageal tumor using NIR and ICG. (**a**) Robotic minimally invasive resection of the tumor. (**b**) Visualization of the tumor during robotic resection using ICG and NIR.

**Figure 3:**
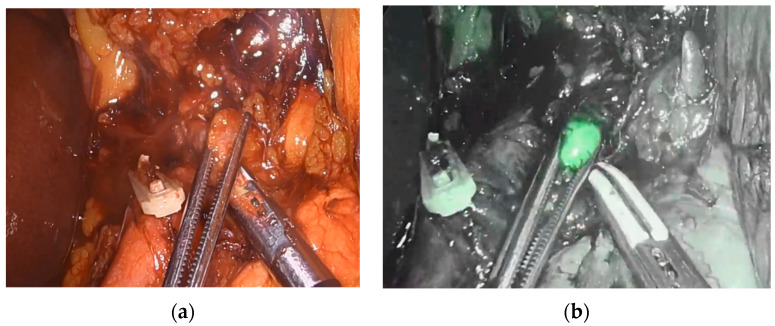
Visualization and identification of a retropancreatic lymph node during abdominal lymph adenectomy. (**a**) Resection of retropancreatic lymph node. (**b**) Visualization of ICG-positive retropancreatic lymph node.

**Figure 4:**
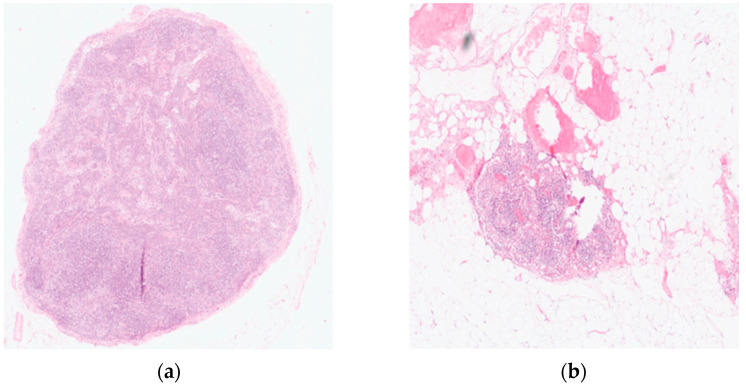
Histopathological workup of a resected lymph node using Hematoxylin and Eosin (HE) staining. (**a**) Tumor-free lymph node (**b**) Lymph node metastases showing a complete response after neoadjuvant treatment. Original magnification 200×.

Endoscopic injection of ICG around the tumor was performed on the day prior to surgery in 18 patients. Two patients underwent the intervention on the day of surgery. No side effects from ICG or endoscopic intervention were reported. Real-time NIR was successfully performed in all patients. Figure 2 and Figure 3 show how ICG visualized and identified the tumor and lymph nodes. One ICG-negative lymph node was resected from the D2 compartment for every patient. In addition, for 15 patients, an ICG-positive lymph node was resected from the D2 compartment and sent to pathology for separate analysis. In 5 patients, no ICG uptake was noted within the D2 compartment, and therefore no extra lymph node was resected. Surgeries performed under the ESOMAP protocol were found to be significantly shorter compared to the RAMIE cohort. [ESOMAP mean 341 min (range 235–486 min) compared to 375 min (range 273–615), *p* = 0.0410]. In particular, the thoracic part of the RAMIE procedure was shorter under the ESOMAP protocol, with a mean time of 151 min (range 90–240) in the ESOMAP group and a mean of 184.4 min (range 90–317) in the RAMIE group, *p* = 0.0198. A mean of 32.9 lymph nodes were resected in the ESOMAP group (range 17–57) compared to a mean of 36.8 (range 14–97) in the RAMIE group, *p* = 0.2384. A total of 15 ICG-positive lymph nodes were resected amongst the study population, of which none showed cancer infiltration. Furthermore, no ICG-negative lymph node showed viable cancer cells. From the remaining lymph nodes that were not analyzed in accordance with their ICG status, *n* = 20 showed tumor infiltration. An example of a histopathological workup is shown in Figure 2. The text continues here (Figure 4).

### 3.2. Postoperative Outcome

Postoperative complications, categorized using the Dindo Clavien classification, are shown in Table 2. In addition, the number of patients with an anastomotic leak is shown. No difference was seen during the length of postoperative stay (ESOMAP: median 12 days, range 10–52 days vs. RAMIE: median 13 days, range 10–123), *p* = 0.9669. In addition, no significant difference was seen in ICU stay, which was a median of 2 days in the ESOMAP group (range 2–16 days), compared to a median of 2 days (range 1–65 days), *p* = 0.6520.

## 4. Discussion

The ESOMAP protocol proved feasibility and safety for the application of NIR using ICG during RAMIE. No statistically significant difference was seen in baseline characteristics or postoperative outcomes for patients under the ESOMAP protocol compared to our standardized RAMIE cohort. Fifty percent of patients in both groups were discharged with no postoperative complications, emphasizing the positive impact of a minimally invasive robotic approach at a high-volume center, especially when comparing this to previously published benchmarks of esophageal cancer surgery [17].

Interestingly, even though additional steps are needed when using different imaging modes, the ESOMAP protocol did not increase the duration of the surgical case. Cases performed under the ESOMAP protocol were significantly shorter than other RAMIE cases at our center, especially for the thoracic part. It must be assumed that the shorter operative time is primarily assigned to a bias, as all patients under the ESOMAP protocol were operated on by a single surgeon, whereas learning, training, and teaching effects might have been present in the RAMIE group. It can be speculated that the ESOMAP protocol facilitates surgical dissection due to the enhanced reality of the surgical image and therefore shortens OR time. As this was a feasibility study, neither result was considered when performing lymphadenectomy. In all cases, a standardized lymphadenectomy was performed, and no compromises or assumptions due to the study protocol were made. Additional lymphadenectomy was only performed if ICG uptake was noted. A follow-up of patients in the future will show the impact of this study on the oncological outcome.

The method of lymph node mapping using near-infrared fluorescent light and ICG has been shown to be feasible before. Park et al. included 29 patients with SCC and a tumor stage of cT1 in their analysis. A robotic-assisted McKeown esophagectomy and total mediastinal lymphadenectomy were performed. The study proved the feasibility and safety of lymph node mapping of the recurrent laryngeal nerve node. It identified unseen metastases (in previous diagnostics) in 20% of patients [16]. However, patients with neoadjuvant treatment or suspected lymph node metastases were excluded, limiting the validity of the outcomes, especially in everyday clinical routines. We consciously included patients with AC and SCC that underwent primary surgery and those who previously completed neoadjuvant treatment to prove feasibility for a majority of patients, as most ESOMAP patients completed a neoadjuvant treatment (95% of patients).

Schlottmann et al. performed peritumoral ICG injection in 9 patients on the day of their scheduled Ivor Lewis esophagectomy, and intraoperative abdominal lymph node mapping was successfully performed in all patients. Interestingly, in line with our findings, most patients did not present with any lymph node metastases (N0) [9]. Even though no selection algorithm was applied prior to surgery, 70% of our included patients had no lymph node metastases (N0) in final pathology, limiting the chance for the tested method to show its potential. Previous analyses have shown that the N stage cannot be reliably predicted by EUS or CT/PET scans, limiting the ability to actively in- or exclude patients with lymph node metastases in a study [18,19]. The following and ongoing multicenter CARDIA trial will enhance the discussion.

Another prospective trial from Osterkamp et al. showed the impact of intraoperative ICG lymphography during the robotic-assisted resection of GEJ cancer. In line with our protocol, NIR was used for a quality check after the completion of the resection. If ICG-positive tissue was found, additional resection was performed. Interestingly, matching our results, none of this ICG-positive tissue showed tumor infiltration, questioning the correlation between ICG visualization and lymph node positivity. We agree with the authors that it is still up for discussion if ICG has the potential to enhance the oncological quality of the resection [20].

A systematic review and meta-analysis by Jimenez-Lillo et al. focusing on the performance of ICG for sentinel lymph node mapping (SLNM) in esophageal cancer revealed six studies with a total of *n* = 65 patients undergoing surgery for esophageal cancer following the performance of ICG imaging for SLNM. Overall, a sensitivity for ICG-detected LNM of 84% (detected nodes by ICG also positive for metastases) and a specificity for ICG-detected LNM of 15% (non-detected nodes by ICG also negative for metastases) was found [21]. While we agree with the author that it is absolutely not an option to diminish surgical treatment in case of a negative SLNM, our study showed the opposite result. As no ICG-negative lymph node showed cancer infiltration, 100% specificity was achieved in our study.

As only feasibility trials with a small number of patients have been published to date, the optimal latency between ICG injection and lymph node visualization remains unclear. ICG injection is typically performed between 15 min and up to three days prior to the surgical intervention in the literature [3,4,9,10,16,21]. We proved that lymph node mapping using NIR and ICG is feasible when a peritumoral injection is performed directly prior to or on the day before surgery, and no differences between methods were seen in our cohort.

The quantification of ICG in resected lymph nodes during esophagectomy has not been performed yet. However, the concept of ICG quantification and use of this as an objective value has been shown before, especially in studies using ICG as an evaluation tool for perfusion, i.e., of the gastric conduit. Flow speed or flow time was recently used as a way of creating an objective way of measurement. Using a digital clock, Slooter et al., for example, analyzed the time to fluorescence enhancement in the right lung when a McKeown procedure was performed or at the base of the gastric conduit for an Ivor Lewis procedure, both at the planned anastomotic site and at the tip of the gastric conduit. The difference between time points was calculated, and a correlation between time to fluorescent enhancement and anastomotic leakage was found [22]. Yukaya et al. used a different approach and recorded ICG visualization of gastric conduit perfusion by applying video analysis software to quantify the change of color during the measurement [23]. Using a porcine model and following the same concept, Nerup et al. developed a software tool that successfully quantified ICG perfusion in a region of interest [24]. A following study from the same group applied the developed method in patients that underwent esophagectomy and found the technique feasible and usable for both a minimally invasive and an open setting [7]. Okubo et al. applied a similar technique to identify sentinel lymph nodes in early gastric cancer [25]. While those studies show promising results, utilization of those methods in esophageal lymph node mapping has yet to be performed. In addition, artificial intelligence has recently been shown to have the ability to identify visual aspects of recorded videos which may provide an automated, reliable, objective, and even self-learning technique applicable to ICG quantification [26,27]. Future analysis of our resected lymph nodes will focus on the true quantification of ICG analyzed through pathology using targeted tools. Furthermore, using ex vivo fluorescence imaging, a correlation between a specimen’s quantitative ICG fluorescence, the amount of ICG accumulated in the lymph node as determined by pathology, and cancer infiltration can be analyzed.

Limitations of this study include the controlled usage of ICG during this feasibility trial as the detergent has only been cleared for intravenous injection yet. FDA or CE approval for other applications has not yet been performed. No adverse events were noted in our cohort from the application of ICG itself, the intervention, or NIR, as is consistent with previous studies. Furthermore, the application of ICG is based on visual perception. While ICG uptake was visualized using NIR, lymph nodes are often not identified as such and attached to the main specimen. If those lymph nodes are further ICG negative, there is no way to tell during the surgery that a lymph node was resected. Hence, a separate statement on ICG visualization cannot be made as it is, yet, solely based on a visual scale

Technically, we noted the robotic NIR fluorescence system used offers fewer modes to visualize ICG when compared to other available laparoscopic tools on the market. Upcoming innovations will hopefully offer even more imaging and quantification tools. Lastly, only a small number of patients at a single institution were included in the study for this pilot trial in RAMIE patients.

## 5. Conclusions

RAMIE can be safely combined with a near-infrared fluorescent imaging protocol to detect lymph node metastases. Further analyses in our center will investigate the ICG-positive tissue, and long-term follow-up data will be analyzed. The application of artificial intelligence tools for quantifying ICG in esophageal lymph node mapping may improve the accuracy of our protocol in the future. After this successful proof-of-concept, a prospective study should be implemented, including more lymph node stations systematically.

## Figures and Tables

**Table 1:** Demographic and oncological data of patients that underwent a RAMIE procedure with and without the ESOMAP protocol.

	ESOMAP	RAMIE	
	Total (%)	Total (%)	*p* Value
Patients	20 (100)	86 (100)	-
Male	17 (85)	71 (82.6)	1
Adenocarcinoma	19 (95)	66 (76.7)	0.1150
Squamous Cell Carcinoma	1 (5)	19 (22.1)	0.1127
Other	0	1 (1.2)	1
Neoadjuvant Therapy			
None	1 (5)	17 (19.8)	0.1849
CROSS	12 (60)	38 (44.2)	0.2234
FLOT	7 (35)	30 (34.9)	1
Other	0	1 (1.2)	1
Pathological Tumor Stage			
T0	3 (15)	14 (16.3)	1
T1a	2 (10)	7 (8.1)	0.6768
T1b	4 (20)	12 (14)	0.4971
T2	4 (20)	11 (12.8)	0.4761
T3	7 (35)	37 (43)	0.6179
T4	0	4 (4.7)	1
Tx	0	1 (1.2)	1
Pathological Nodal Stage			
N0	14 (70)	48 (55.8)	0.3170
N1	2 (10)	17 (19.8)	0.5174
N2	3 (15)	10 (11.6)	0.7079
N3	1 (5)	11 (12.8)	0.4561

**Table 2:** Postoperative complications of patients that underwent a RAMIE procedure compared to patients that underwent the ESOMAP protocol in addition to RAMIE.

	ESOMAP	RAMIE	
	Total (%)	Total (%)	*p* Value
Patients	20 (100)	86 (100)	-
Postoperative complications			
None	10 (50)	42 (48.8)	1
Clavien Dindo I	0	3 (3.5)	1
Clavien Dindo II	2 (10)	4 (4.6)	0.3161
Clavien Dindo IIIa	5 (25)	24 (27.9)	1
Clavien Dindo IIIb	2 (10)	4 (4.6)	0.3161
Clavien Dindo Iva	1 (5)	6 (7)	1
Clavien Dindo Ivb	0	1 (1.2)	1
Clavien Dindo V	0	2 (2.4)	1
Anastomotic Leak			
Type I	0	0	1
Type II	1 (5)	9 (10.5)	0.6835
Type III	1 (5)	2 (2.3)	0.4695

## Data Availability

The data presented in this study are available on request from the corresponding author. The data are not publicly available due to further ongoing analysis.

## References

[1] Hiranyatheb P., Osugi H. (2015). Radical lymphadenectomy in esophageal cancer: From the past to the present. Dis. Esophagus.

[2] Hölscher A.H., Law S. (2020). Esophagogastric junction adenocarcinomas: Individualization of resection with special considerations for Siewert type II, and Nishi types EG, E=G and GE cancers. Gastric Cancer.

[3] Hachey K.J., Gilmore D.M., Armstrong K.W., Harris S.E., Hornick J.L., Colson Y.L., Wee J.O. (2016). Safety and feasibility of near-infrared image-guided lymphatic mapping of regional lymph nodes in esophageal cancer. J. Thorac. Cardiovasc. Surg..

[4] Schaafsma B.E., Mieog J.S., Hutteman M., van der Vorst J.R., Kuppen P.J., Löwik C.W., Frangioni J.V., van de Velde C.J., Vahrmeijer A.L. (2011). The clinical use of indocyanine green as a near-infrared fluorescent contrast agent for image-guided oncologic surgery. J. Surg. Oncol..

[5] Xiong L., Gazyakan E., Yang W., Engel H., Hünerbein M., Kneser U., Hirche C. (2014). Indocyanine green fluorescence-guided sentinel node biopsy: A meta-analysis on detection rate and diagnostic performance. Eur. J. Surg. Oncol. (EJSO).

[6] Thammineedi S.R., Patnaik S.C., Saksena A.R., Ramalingam P.R., Nusrath S. (2020). The Utility of Indocyanine Green Angiography in the Assessment of Perfusion of Gastric Conduit and Proximal Esophageal Stump Against Visual Assessment in Patients Undergoing Esophagectomy: A Prospective Study. Indian J. Surg. Oncol..

[7] Nerup N., Svendsen M.B.S., Svendsen L.B., Achiam M.P. (2020). Feasibility and usability of real-time intraoperative quantitative fluorescent-guided perfusion assessment during resection of gastroesophageal junction cancer. Langenbeck’s Arch. Surg..

[8] Vecchiato M., Martino A., Sponza M., Uzzau A., Ziccarelli A., Marchesi F., Petri R. (2020). Thoracic duct identification with indocyanine green fluorescence during minimally invasive esophagectomy with patient in prone position. Dis. Esophagus.

[9] Schlottmann F., Barbetta A., Mungo B., Lidor A.O., Molena D. (2017). Identification of the Lymphatic Drainage Pattern of Esophageal Cancer with Near-Infrared Fluorescent Imaging. J. Laparoendosc. Adv. Surg. Tech..

[10] Hosogi H., Yagi D., Sakaguchi M., Akagawa S., Tokoro Y., Kanaya S. (2021). Upper mediastinal lymph node dissection based on mesenteric excision in esophageal cancer surgery: Confirmation by near-infrared image-guided lymphatic mapping and the impact on locoregional control. Esophagus.

[11] Müller D.T., Babic B., Herbst V., Gebauer F., Schlößer H., Schiffmann L., Chon S.H., Schröder W., Bruns C.J., Fuchs H.F. (2020). Does Circular Stapler Size in Surgical Management of Esophageal Cancer Affect Anastomotic Leak Rate? 4-Year Experience of a European High-Volume Center. Cancers.

[12] Fuchs H.F., Harnsberger C.R., Broderick R.C., Chang D.C., Sandler B.J., Jacobsen G.R., Bouvet M., Horgan S. (2017). Simple preoperative risk scale accurately predicts perioperative mortality following esophagectomy for malignancy. Dis. Esophagus.

[13] Fuchs H.F., Müller D.T., Leers J.M., Schröder W., Bruns C.J. (2019). Modular step-up approach to robot-assisted transthoracic esophagectomy-experience of a German high volume center. Transl. Gastroenterol. Hepatol..

[14] Leers J.M., Knepper L., van der Veen A., Schröder W., Fuchs H., Schiller P., Hellmich M., Zettelmeyer U., Brosens L.A.A., Quaas A. (2020). The CARDIA-trial protocol: A multinational, prospective, randomized, clinical trial comparing transthoracic esophagectomy with transhiatal extended gastrectomy in adenocarcinoma of the gastroesophageal junction (GEJ) type II. BMC Cancer.

[15] Hölscher A.H., Gockel I., Porschen R. (2019). Updated German S3 guidelines on esophageal cancer and supplements from a surgical perspective. Chirurg.

[16] Park S.Y., Suh J.W., Kim D.J., Park J.C., Kim E.H., Lee C.Y., Lee J.G., Paik H.C., Chung K.Y. (2018). Near-Infrared Lymphatic Mapping of the Recurrent Laryngeal Nerve Nodes in T1 Esophageal Cancer. Ann. Thorac. Surg..

[17] Schmidt H.M., Gisbertz S.S., Moons J., Rouvelas I., Kauppi J., Brown A., Asti E., Luyer M., Lagarde S.M., Berlth F. (2017). Defining Benchmarks for Transthoracic Esophagectomy: A Multicenter Analysis of Total Minimally Invasive Esophagectomy in Low Risk Patients. Ann. Surg..

[18] Thosani N., Singh H., Kapadia A., Ochi N., Lee J.H., Ajani J., Swisher S.G., Hofstetter W.L., Guha S., Bhutani M.S. (2012). Diagnostic accuracy of EUS in differentiating mucosal versus submucosal invasion of superficial esophageal cancers: A systematic review and meta-analysis. Gastrointest. Endosc..

[19] van Vliet E.P., Heijenbrok-Kal M.H., Hunink M.G., Kuipers E.J., Siersema P.D. (2008). Staging investigations for oesophageal cancer: A meta-analysis. Br. J. Cancer.

[20] Osterkamp J., Strandby R., Nerup N., Svendsen M.B., Svendsen L.B., Achiam M. (2023). Intraoperative near-infrared lymphography with indocyanine green may aid lymph node dissection during robot-assisted resection of gastroesophageal junction cancer. Surg. Endosc..

[21] Jimenez-Lillo J., Villegas-Tovar E., Momblan-Garcia D., Turrado-Rodriguez V., Ibarzabal-Olano A., De Lacy B., Diaz-Giron-Gidi A., Faes-Petersen R., Martinez-Portilla R.J., Lacy A. (2021). Performance of Indocyanine-Green Imaging for Sentinel Lymph Node Mapping and Lymph Node Metastasis in Esophageal Cancer: Systematic Review and Meta-Analysis. Ann. Surg. Oncol..

[22] Slooter M.D., de Bruin D.M., Eshuis W.J., Veelo D.P., van Dieren S., Gisbertz S.S., van Berge Henegouwen M.I. (2021). Quantitative fluorescence-guided perfusion assessment of the gastric conduit to predict anastomotic complications after esophagectomy. Dis. Esophagus.

[23] Yukaya T., Saeki H., Kasagi Y., Nakashima Y., Ando K., Imamura Y., Ohgaki K., Oki E., Morita M., Maehara Y. (2015). Indocyanine Green Fluorescence Angiography for Quantitative Evaluation of Gastric Tube Perfusion in Patients Undergoing Esophagectomy. J. Am. Coll. Surg..

[24] Nerup N., Andersen H.S., Ambrus R., Strandby R.B., Svendsen M.B.S., Madsen M.H., Svendsen L.B., Achiam M.P. (2017). Quantification of fluorescence angiography in a porcine model. Langenbeck’s Arch. Surg..

[25] Okubo K., Uenosono Y., Arigami T., Matsushita D., Yanagita S., Kijima T., Amatatsu M., Ishigami S., Maemura K., Natsugoe S. (2018). Quantitative assessment of fluorescence intensity of ICG in sentinel nodes in early gastric cancer. Gastric Cancer.

[26] Garrow C.R., Kowalewski K.F., Li L., Wagner M., Schmidt M.W., Engelhardt S., Hashimoto D.A., Kenngott H.G., Bodenstedt S., Speidel S. (2021). Machine Learning for Surgical Phase Recognition: A Systematic Review. Ann. Surg..

[27] Hashimoto D.A., Rosman G., Witkowski E.R., Stafford C., Navarette-Welton A.J., Rattner D.W., Lillemoe K.D., Rus D.L., Meireles O.R. (2019). Computer Vision Analysis of Intraoperative Video: Automated Recognition of Operative Steps in Laparoscopic Sleeve Gastrectomy. Ann. Surg..

